# Exploring the factors influencing the health-related quality of life in patients experiencing adverse drug reactions: a cross-sectional study

**DOI:** 10.1186/s41687-024-00790-0

**Published:** 2024-09-27

**Authors:** Garapati Pavan, Manish Kumar, Krishna Murti, Sameer Dhingra, V. Ravichandiran

**Affiliations:** 1 Department of Pharmacy Practice, National Institute of Pharmaceutical Education and Research, Hajipur, India; 2 Department of Pharmacology, Indira Gandhi Institute of Medical Sciences, Patna, India; 3 Adverse Drug Reaction Monitoring Center, Indira Gandhi Institute of Medical Sciences, Patna, India; 4 Adverse Drug Reaction Monitoring Center, National Institute of Pharmaceutical Education and Research, Hajipur, India

**Keywords:** ADRs, HRQoL, EQ-5D-5L, ADR severity, QoL

## Abstract

**Background:**

This study aimed to assess the factors influencing health-related quality of life (HRQoL) in patients experiencing adverse drug reactions (ADRs) at a tertiary care public sector hospital. A cross-sectional study was conducted over a period of 18 months, and included both male and female patients aged 18 years and above. Patients who visited the outpatient and inpatient departments with complaints associated with ADRs were included in this study. HRQoL data were collected using the EuroQol—5 Dimension—5 Level (EQ-5D-5L) questionnaire to assess five dimensions of health on a five-level scale. Descriptive statistics, t-tests, and analysis of variance were used to analyze the data. Multivariate regression analysis was performed to identify the potential determinants of HRQoL.

**Results:**

A total of 316 patients were included in the study among these participants, of which 54% were female, and 65% were from rural areas. The majority (68%) of the patients had moderately severe ADRs, and 63% of the participants had an income < 2.5 lakh Indian rupees (3009 USD). The mean EQ-5D-5L and EuroQoL Visual Analog Scale (EQ VAS) scores of the study participants were 0.714 and 69.73, respectively. The variables ADR severity, income, and age showed a significant difference (*p* < 0.05) in HRQoL.

**Conclusion:**

This study provides insights into HRQoL among patients with ADRs and identifies the determinants of HRQoL. The findings of this study will contribute to improving patient-centered care and optimizing patient outcomes.

## Background

Adverse drug reactions (ADRs) are harmful and unintended effects of medications that occur during regular clinical use, and they can range from minor side effects to life-threatening events [1]. Literature shows 5–10% of ADRs were responsible for hospital admissions [2, 3]. In developed countries, the prevalence of hospital admissions due to ADRs was 6.3% and in developing countries 5.5% [4]. A study from India reported prevalence of ADRs was 3.7% and 0.7% ADRs were responsible for hospitalization [5]. The estimated economic burden incurred due to ADRs in Europe at €79 billion [6] and in US at $30.1 billion [7]. A study from India reported direct and indirect costs associated with ADR management were €3008 and €27,206 respectively [8].

ADRs can significantly impact health-related quality of life (HRQoL), leading to physical, emotional, and social burdens. Patients with ADRs may experience pain, fatigue, swelling, and numbness affecting their ability to perform daily activities which cause physical discomfort [9]. The experience of dealing with skin ADRs affect patients’ self-esteem and impacting emotional health leading to anxiety or depression [9, 10]. The ADRs affecting skin impacts person’s ability to interact with others leading to social avoidance [11]. Additionally, treating ADRs can be costly and time-consuming, leading to further stress and burden on patients and their families [12, 13].

Quality of life (QoL) measures an individual’s perception of their physical, mental, and social well-being concerning their health status and the treatments they receive. HRQoL is affected by various factors, including the severity and duration of illnesses, disabilities, and chronic conditions, including social and environmental factors [14, 15]. Improving HRQoL is a priority for healthcare providers, as it can impact individuals’ overall health outcomes and their ability to participate in daily activities [16]. HRQoL is relevant worldwide and is increasingly being studied and measured around the globe.

HRQoL is often measured using standardised questionnaires or surveys that ask individuals about their ability to perform activities of daily living, their emotional well-being, their social relationships, and their overall satisfaction with their health status and healthcare [17]. These measurements can provide valuable information for healthcare providers, policymakers, and researchers to assess the impact of various interventions and treatments on individuals’ QoL [14, 15].

The availability of ADR-induced QoL data may vary depending on several factors, including the specific drugs and health conditions considered [18]. However, in many countries, including India, there is often a lack of robust and systematic collection of ADR-induced QoL data. In India, the primary focus of pharmacovigilance efforts has been on monitoring and reporting adverse drug reactions rather than specifically assessing their impact on QoL. The Pharmacovigilance Programme of India (PvPI) is a government initiative that aims to collect, analyze, and monitor ADRs under the Indian Pharmacopoeia Commission (IPC) [19]. While the programme helps to identify and document adverse reactions; however, it may not consistently capture data on the impact of these reactions on patients’ QoL.

The existing literature highlights a dearth of data on the determinants of HRQoL among individuals with ADRs. Therefore, this study sought to fill this gap by investigating the factors affecting QoL in patients with ADRs. The EuroQol—5 Dimension—5 Level (EQ-5D-5L) questionnaire was used to comprehensively evaluate various QoL dimensions. The EQ-5D-5L is known for its ease of use and the calculation of quality-adjusted life years (QALYs). The QALYs generated from this study results can be used for the cost-effectiveness analysis of any new intervention focusing on ADR management or prevention.

## Methods

### Study design

A cross-sectional questionnaire-based study was conducted to assess QoL among patients who experienced ADRs at a tertiary care public sector hospital. The study was conducted over 18 months from December 2021 to June 2023.

### Study setting and participants

Bihar is located in the eastern part of India with more than 100 million population and is India’s third most populous state. Bihar is classified as a low-income state and is one of India’s less economically developed states [20]. The primary language spoken is Hindi, however, several regional languages and dialects are also prevalent, including Bhojpuri, Maithili, and Magahi. About 11.3% of the population lives in urban areas, indicating a predominantly rural population. According to the NITI Aayog Health Index ranking, Bihar has been ranked last among the large states [21]. The state has inadequate health care infrastructure, including shortage of beds and health care professionals.

This study was conducted at the Indira Gandhi Institute of Medical Sciences (IGIMS), Patna, a tertiary care public sector hospital that provides health services to the people of Bihar and nearby regions. Additionally, IGIMS is an ADR monitoring center (AMC) working under IPC-PvPI. The hospital accommodates 1163 beds and during 2022–2023 a total of 42,529 in-patients and 8,47,493 outpatients treated at IGIMS. More than half of the patients utilising this hospital belong below the poverty line and come from rural areas.

Patients with ADRs attending the inpatient and outpatient departments of IGIMS were identified by health care professionals and referred to the AMC for reporting ADRs to the IPC. Eligible patients were enrolled in the study based on predefined inclusion and exclusion criteria. The study participants included patients of both sexes, aged 18 years and above, who experienced ADRs. Patients with intellectual disabilities, physical handicaps, or ADRs due to intentional drug abuse were excluded from the study. Participants with incomplete information were excluded to ensure the accuracy and integrity of the data. Eligible participants were approached, provided with a detailed explanation of the study, and informed consent was obtained prior to their inclusion in the study.

### Ethical considerations

The study adhered to ethical guidelines and obtained the necessary ethical approval from the institutional ethics committee of IGIMS, Patna, Bihar, India (373/IEC/IGIMS/2021). The participants’ privacy, confidentiality, and rights were protected throughout the study. Adequate measures were taken to ensure informed consent, voluntary participation, and the right to withdraw from the study.

### Data collection

A trained individual administered the questionnaire through face-to-face interviews or self-administration, depending on the participant’s preference and feasibility. The interviews were conducted the same day after the patients were included in the study. All interviews took place in the hospital, and during the interviews, the patients were accompanied by their caregivers or relatives. A structured questionnaire was used to collect demographic information, medical history, and details of ADRs experienced by the participants. While collecting information regarding medical history sometimes patient caregivers responded on behalf of the patients when the patients found it difficult to recall the necessary details. Based on the Hartwig’s criteria ADRs were classified as mild, moderate or severe. Mild ADRs cause minimal discomfort and are self-limiting in nature. Moderate ADRs have a clinical impact and require treatment to manage them. Severe ADRs have a significant clinical impact and require immediate medical intervention [22].

The EQ-5D-5L Hindi questionnaire was administered to assess the participants’ QoL. The EQ-5D-5L consisted of five dimensions mobility, self-care, usual activities, pain/discomfort, and anxiety/depression. Each dimension contains five response levels: no problems, slight problems, moderate problems, severe problems, and extreme problems. The EuroQol Visual Analog Scale (EQ VAS) is a self-reported measure that assesses an individual’s overall health or quality of life on a continuum from 0 to 100, with 0 indicating the worst imaginable health state and 100 representing the best possible health state [23–25]. The QoL assessment using the EQ-5D-5L was performed at a single time point, specifically during the occurrence of the ADR. The EQ-5D-5L questionnaire has been widely used, validated translated and culturally adapted for use across the globe, including India. This study used an Indian value set and algorithm to calculate the utility score [26, 27].

### Data analysis

The collected data were entered into the Statistical Package for the Social Science (SPSS) software and descriptive statistical analysis was performed to summarise the demographic characteristics, ADR severity, and QoL scores obtained from the EQ-5D-5L. The distribution of participants across the response levels of the EQ-5D-5L was examined to assess the proportion of patients experiencing problems in each dimension. Descriptive information was presented as frequencies and percentages. To investigate differences in QoL scores between the two groups, Student’s t-test was employed. For the comparisons involving more than two groups, analysis of variance (ANOVA) was performed. Multivariate regression models were employed to identify potential determinants of QoL. A *p* value of < 0.05 was considered statistically significant. The statistical analyses were conducted using SPSS version 25.

## Results

A total of 320 patients were initially enrolled in the study. Four participants were excluded from the analysis due to missing information, resulting in a final sample size of 316 participants.

The mean age of the participants was 41.74 years, with a standard deviation (SD) of 16.83. Among the 316 participants, 54.1% were female. Table 1 presents the sociodemographic and clinical characteristics of the participants. The largest proportion (37%) of the respondents belonged to the 31–50 years age group. In terms of residence, 65.2% of the participants were from rural areas, and 75.3% were married. Regarding education, approximately one-third (34.8%) of the respondents had completed their secondary education. The annual income measured in terms of Indian Rupees (INR) where, one lakh equals to hundred thousand. Over half (55.1%) of the participants fell into the 1–2.5 lakh INR (1203-3009USD) income category. Furthermore, the majority of the ADRs (68.04%) were classified as moderately severe. A large proportion (60.8%) of the participants were from in-patient wards and 33.5% of the ADRs were related to the antibiotic class of drugs. Over 40% of the participants were from the general medicine and medical oncology departments.

**Table 1:** Participants’ sociodemographic and clinical characteristics

Variable	Frequency (%)
*Age*	
18–30	97 (30.7)
31–50	117 (37)
> 50	102 (32.3)
*Gender*	
Male	145 (45.9)
Female	171 (54.1)
*Marital state*	
Single	78 (24.7)
Married	238 (75.3)
*Residence*	
Urban	110 (34.8)
Rural	206 (65.2)
*Education*	
Illiterate	101 (32)
Primary	27 (8.5)
Secondary	110 (34.8)
Graduation and more	78 (24.7)
*Income in lakh INR*	
< 1	27 (8.5)
1–2.5	174 (55.1)
2.5–5	99 (31.3)
> 5	16 (5.1)
*Occupation*	
Daily wage earner	31 (9.8)
Farmer	41 (13)
Employee	43 (13.6)
Unemployed	56 (17.7)
Student	52 (16.5)
Homemaker	93 (29.4)
*ADR severity*	
Mild	49 (15.5)
Moderate	215 (68.0)
Severe	52 (16.5)
Category of participants	
In-patient	192 (60.8)
Out-patient	124 (39.2)
*Department*	
General medicine	69 (21.8)
Medical oncology	63 (19.9)
General surgery	26 (8.2)
Neuro medicine	22 (7.0)
Orthopaedic	25 (7.9)
Skin and VD	17 (5.4)
Gastroenterology	11 (3.5)
ENT	11 (3.5)
Others	72 (22.8)
*Drugs responsible for ADRs*	
Antibiotics	105 (33.5)
Taxanes	23 (7.3)
Alkylating agents	16 (5.1)
NSAIDs	11 (3.5)
β-blockers	8 (2.5)
Others	151 (48.1)

*INR* Indian rupee (one lakh equals to hundred thousand), *VD* venereal diseases, *ENT* ear nose throat, *NSAIDs* non−steroidal anti−inflammatory drugs, *% *percentage

Table 2 represents the distribution of HRQoL across five dimensions and five levels. Most of the participants had no problems with mobility (47.5%) and anxiety/depression (56.3%). Most of the respondents had slight problems with selfcare (44.9%), usual activity (60.4%) and pain/discomfort (44.6%).

**Table 2:** Descriptive distribution of EQ-5D-5L responses provided by the participants

Dimensions and level of problem	Mobility	Selfcare	Usual activity	Pain/discomfort	Anxiety/depression
	*n* (%)	*n* (%)	*n* (%)	*n* (%)	*n*(%)
No problem	150 (47.5)	122 (38.6)	49 (15.5)	7 (2.2)	178 (56.3)
Slight problem	114 (36.1)	142 (44.9)	191 (60.4)	141 (44.6)	95 (30.1)
Moderate problem	41 (13.0)	43 (13.6)	63 (19.9)	134 (42.4)	28 (8.9)
Severe problem	10 (3.2)	7 (2.2)	11 (3.5)	29 (9.2)	14 (4.4)
Extreme problem	1 (0.3)	2 (0.6)	2 (0.6)	5 (1.6)	1 (0.3)

*n* frequency, *%* percentage

In Table 3, the association between QoL scores and variables is presented. The mean HRQoL scores for the EQ-5D-5L and EQ VAS were 0.71 and 69.73, respectively. Participants aged over 50 years had significantly (*p* < 0.001) lower QoL scores than the other age groups. The following variables showed a significant (*p* < 0.001) association with utility and EQ VAS scores: education, occupation, and ADR severity. Females had a higher utility score (0.73) than males additionally, single participants had a healthier score (0.80) than married individuals. A significant (*p* < 0.001) difference in EQ VAS scores was observed among respondents with low income.

**Table 3:** Association between HRQoL scores and sociodemographic and clinical characteristics

Variable	Utility score	*p* value	EQ VAS score	*p* value
	Mean ± SD		Mean ± SD	
*Age*		< 0.001^*^		< 0.001^*^
18–30	0.783 ± 0.213		75.81 ± 8.35	
31–50	0.696 ± 0.236		70.44 ± 8.30	
> 50	0.620 ± 0.325		63.15 ± 10.24	
*Gender*		0.002^*^		0.101
Male	0.694 ± 0.317		69.43 ± 11.35	
Female	0.731 ± 0.222		69.99 ± 9.32	
*Marital state*		0.005^*^		0.484
Single	0.802 ± 0.162		76.41 ± 8.37	
Married	0.686 ± 0.292		67.55 ± 9.92	
*Residence*		0.454		0.736
Urban	0.739 ± 0.263		70.23 ± 9.48	
Rural	0.701 ± 0.274		68.51 ± 10.51	
*Education*		0.028^*^		< 0.001^*^
Illiterate	0.663 ± 0.268		64.74 ± 8.98	
Primary	0.649 ± 0.369		67.59 ± 14.93	
Secondary	0.741 ± 0.237		71.73 ± 8.87	
Graduation and more	0.765 ± 0.266		74.13 ± 9.07	
*Income in lakh INR*		0.075		< 0.001^*^
< 1	0.593 (0.391)		61.30 ± 15.07	
1–2.5	0.716 (0.274)		70.18 ± 9.34	
2.5–5	0.734 (0.229)		71.53 ± 9.55	
> 5	0.779 (0.163)		68.00 ± 8.46	
*Occupation*		< 0.001^*^		< 0.001^*^
Daily wage earner	0.764 ± 0.207		69.74 ± 7.88	
Farmer	0.624 ± 0.388		65.41 ± 13.27	
Employee	0.781 ± 0.247		72.70 ± 8.98	
Unemployed	0.605 ± 0.313		65.43 ± 9.09	
Student	0.808 ± 0.157		78.15 ± 7.72	
Homemaker	0.720 ± 0.227		66.31 ± 8.95	
*ADR severity*		< 0.001^*^		< 0.001^*^
Mild	0.837 ± 0.148		73.29 ± 10.47	
Moderate	0.766 ± 0.155		71.01 ± 8.23	
Severe	0.384 ± 0.439		61.10 ± 13.08	
Total	0.714 ± 0.270	NA	69.73 ± 10.29	NA

*SD* standard deviation, *EQ VAS* EuroQoL visual analogue scale, *INR* Indian rupee (one lakh equals to hundred thousand), * *p* value <0.05, *NA* not applicable

All variables were included in the linear regression analysis, and the values are presented in Table 4. The results indicated that both gender and income significantly (*p* < 0.05) influenced utility and VAS scores. Moreover, the severity of ADRs was found to be a significant (*p* < 0.01) variable affecting the HRQoL scores.

**Table 4:** Determinants of HRQoL among patients with ADRs

	Model	Unstandardised B	Coefficient of standard error	Standardised coefficients beta	t	Significance
Utility Score	Constant	1.110	0.118		9.436	0.000
	Age	−0.042	0.028	−0.123	−1.490	0.137
	Gender	0.059	0.031	0.109	1.915	0.056
	Marital state	−0.076	0.048	−0.121	−1.565	0.119
	Residence	0.016	0.031	0.028	0.516	0.606
	Education	−0.006	0.017	−0.027	−0.370	0.712
	Income	0.068	0.023	0.177	2.973	0.003^*^
	Occupation	0.004	0.009	0.023	0.417	0.677
	Severity	−0.225	0.023	−0.472	−9.736	< 0.001^*^
EQ VAS Score	Constant	85.251	4.268		19.977	0.000
	Age	−4.438	1.023	−0.343	−4.337	< 0.001^*^
	Gender	2.005	1.123	0.097	1.785	0.075
	Marital state	−2.449	1.757	−0.103	−1.393	0.164
	Residence	−0.667	1.120	−0.031	−0.595	0.552
	Education	0.706	0.602	0.081	1.174	0.241
	Income	1.844	0.829	0.126	2.224	0.027^*^
	Occupation	0.138	0.320	0.023	0.431	0.666
	Severity	−5.425	0.840	−0.298	−6.460	< 0.001^*^

* *p* value <0.05, *EQ VAS* EuroQoL visual analogue scale

## Discussion

This study investigated the HRQoL among patients with ADRs using the EQ-5D-5L questionnaire. The findings reveal that the mean utility score (0.714 ± 0.270) was lower than the utility score of the Indian general population (0.849 ± 0.212) [26, 28] but higher than the utility score of cancer patients (0.602 ± 0.311) [29]. Additionally, the mean EQ-VAS score (69.73 ± 10.29) was lower than that of both the Indian general population (75.18 ± 16.42) [26, 28] and cancer patients (75 ± 12.3) [29]. These results indicate that patients with ADRs experience a lower level of HRQoL than the general population. Lower scores suggest a burden of health problems and reduced overall well-being among individuals affected by ADRs.

Over half of the respondents reported problems with mobility which is higher (30.3%) than the Indian general population [26]. Two studies from India reported ADRs responsible for decreased physical domain scores [30, 31], another study reported 25% of the patients with ADRs reported diminished general condition [32] and a study showed lower physical component summary patients with ADRs [33]. Patients with ADRs like muscle pain, dizziness, fatigue and numbness lead to mobility issues. The reported lower physical health among patients indicating noticeable mobility impairment leading to overall diminished QoL. Approximately 30.1% of the respondents reported slight problems related to anxiety or depression which is lower (24.8%) than the Indian general population [26]. A study from India reported patients with ADRs reported diminished psychological health [30], and another study reported a quarter of the participants’ mood or enjoyment of life was impacted [32]. A cross-sectional study reported a lower mental component summary among patients with ADRs [33], and a study revealed depression and anxiety prevalence was higher among ADR patients than the general population [34]. A higher reporting of mobility problems and anxiety/depression is consistent among ADR patients. This highlights the significant impact of ADRs on patients’ QoL and the importance of addressing both physical and mental health aspects in ADR management.

The study findings revealed a significant association (*p* < 0.001) between HRQoL scores and age, indicating that older age is a contributing factor to impaired QoL. Specifically, the HRQoL scores decreased with increasing age, which is consistent with previous studies conducted in India [26, 28, 35], as well as studies conducted in other countries [36, 37]. The consistent findings across different studies and countries further support the robustness and generalisability of the age-related impact on HRQoL. Indeed, the older age group is more susceptible to having multiple diseases and a higher prevalence of mental health issues. Furthermore, mental health issues, such as depression, anxiety, and cognitive decline are more prevalent among older adults [38]. These conditions may further compound the impact of ADRs on the individual’s overall QoL and well-being. Tailored interventions, including medication management, mental health support, and comprehensive care, may help mitigate the impact of ADRs and enhance the overall QoL in this population.

The study findings demonstrated a significant (*p* < 0.05) impact of participants’ income on HRQoL. These results align with previous studies that have also investigated the influence of income on QoL [39, 40]. Indeed, studies have demonstrated a significant association between insufficient income and high anxiety levels [41, 42]. Specifically, the findings indicate that individuals with lower incomes tend to experience a low level of QoL. Low income could pose various challenges and limitations that may negatively affect multiple aspects of a person’s life, including access to healthcare, living conditions, and social support. These factors might collectively contribute to a diminished quality of life.

The study findings indicate that the severity of ADRs significantly (*p* < 0.001) influences HRQoL. These results are consistent with previous studies conducted on various diseases, which have consistently shown that disease severity is associated with QoL [43–45]. When ADRs are more severe, they may have a greater impact on an individual’s physical and psychological well-being, resulting in a lower HRQoL. Higher levels of symptom burden, functional impairment, pain, and discomfort associated with severe ADRs can adversely affect different dimensions of a person’s quality of life. Understanding the impact of ADR severity on HRQoL is essential for healthcare professionals to accurately assess the consequences of adverse reactions and tailor appropriate interventions. By considering the severity of ADRs and their association with QoL, healthcare providers can prioritise effective management strategies, minimise the impact of ADRs on patients’ well-being, and optimise their overall treatment outcomes.

The study results might be useful in developing patient-centred interventions and policy guidelines to improve patient-reported outcomes. The study highlights the importance of considering individual demographics of patients, such as age, income and ADR severity along with treatment plans to provide a personalised approach that may improve patient outcomes. The study results may enable more patient-centred care, improved treatment decision-making, and enhanced drug safety monitoring, ultimately leading to improved healthcare outcomes and better QoL for affected individuals.

The study results were reliable and replicable; however, the study has some limitations. The study was conducted at a public sector hospital, where most of the population came from rural areas. This could impact the QoL scores as access to health care diminished and patients may have had to travel long hours to reach the hospital. The reliance on self-reported measures may have introduced response bias and subjectivity in QoL assessments. Collecting QoL data only once during the occurrence of the ADR may not have captured long-term or post resolution QoL changes. The EQ-5D-5L is a generic scale and may not capture all disease-specific or condition-specific variables. Differences in understanding and interpreting the questionnaire items can lead to variability in responses. This is particularly relevant in populations with varying levels of health literacy and education. Being in a hospital setting can cause stress and anxiety for patients, which might influence their responses to the survey. The presence of caregivers or relatives might influence patients’ responses.

## Conclusion

These findings emphasise the need for interventions and support measures to address the specific challenges faced by the patients and improve their HRQoL. Strategies to improve HRQoL include addressing underlying health conditions, improving access to healthcare, providing social and emotional support, and addressing environmental factors that may impact individuals’ health and well-being. Therefore, healthcare providers need to identify and manage ADRs promptly to improve the QoL of patients and promote better health outcomes.

## Data Availability

The datasets used and/or analysed during the current study are available from the corresponding author on reasonable request.

## References

[1] Aronson JK, Ferner RE (2005) Clarification of terminology in drug safety. Drug safety 28:851–870. 10.2165/00002018-200528100-00003 10.2165/00002018-200528100-00003 16180936

[2] Komagamine J (2024) Prevalence of urgent hospitalizations caused by adverse drug reactions: a cross-sectional study. Sci Rep 14:6058 38480855 10.1038/s41598-024-56855-z PMC10937656

[3] Oscanoa TJ, Lizaraso F, Carvajal A (2017) Hospital admissions due to adverse drug reactions in the elderly. A meta-analysis. Eur J Clin Pharmacol 73:759–770 28251277 10.1007/s00228-017-2225-3

[4] Angamo MT, Chalmers L, Curtain CM, Bereznicki LR (2016) Adverse-drug-reaction-related hospitalisations in developed and developing countries: a review of prevalence and contributing factors. Drug Saf 39:847–857 27449638 10.1007/s40264-016-0444-7

[5] Ramesh M, Pandit J, Parthasarathi G (2003) Adverse drug reactions in a south Indian hospital—their severity and cost involved. Pharmacoepidemiol Drug Saf 12:687–692 14762985 10.1002/pds.871

[6] Commission of the European Communities (2008) Commission staff working document Annex 2 of the report on the impact assessment of strengthening and rationalizing EU Pharmacovigilance. https://health.ec.europa.eu/document/download/bc08b27f-4850-4b1c-9832-bb326d1f68ae_en?filename=pharmacovigilance-ia-vol2_en.pdf. Accessed 1 June 2024

[7] Sultana J, Cutroneo P, Trifirò G (2013) Clinical and economic burden of adverse drug reactions. J Pharmacol Pharmacotherapeutics 4:S73–S77 10.4103/0976-500X.120957 PMC3853675 24347988

[8] Pattanaik S, Dhamija P, Malhotra S, Sharma N, Pandhi P (2009) Evaluation of cost of treatment of drug-related events in a tertiary care public sector hospital in Northern India: a prospective study. Br J Clin Pharmacol 67:363–369 19523017 10.1111/j.1365-2125.2008.03346.x PMC2675048

[9] Du R, Yang H, Zhu J, Zhou H, Ma L, Getu MA, Chen C, Wang T (2022) Experience of patients with lung cancer and with targeted therapy-related skin adverse drug reactions: a qualitative study. Asia-Pacific J Oncol Nurs 9:100115 10.1016/j.apjon.2022.100115 PMC9479359 36118625

[10] Suh HJ, Florez A, Sacristan V, Rodriguez Martinez A, Fernandez F, Vilanova-Trillo L, Constenla M, Pereiro M (2021) Cutaneous adverse events in patients receiving anticancer therapy in a tertiary hospital setting: the old and the new. Int J Dermatol 60:208–216 33502780 10.1111/ijd.15081

[11] Jui-Chun CHAN, Yun-Hsiang LEE, Chien-Ying LIU, Hui-Hsuan SHIH, Pei-Kwei TSAY, Woung-Ru TANG (2019) A correlational study of skin toxicity and quality of life in patients with advanced lung cancer receiving targeted therapy. J Nurs Res 27:e51 10.1097/jnr.0000000000000339 31397829

[12] Sendekie AK, Kasahun AE, Limenh LW, Dagnaw AD, Belachew EA (2023) Clinical and economic impact of adverse drug reactions in hospitalised patients: prospective matched nested case–control study in Ethiopia. BMJ open. 10.1136/bmjopen-2023-073777 37280017 10.1136/bmjopen-2023-073777 PMC10255243

[13] Jordan S, Logan PA, Panes G, Vaismoradi M, Hughes D (2018) Adverse drug reactions, power, harm reduction, regulation and the ADRe profiles. 10.3390/pharmacy6030102. Pharmacy 10.3390/pharmacy6030102 PMC6165166 30231573

[14] Palermo TM, Long AC, Lewandowski AS, Drotar D, Quittner AL, Walker LS (2008) Evidence-based assessment of health-related quality of life and functional impairment in pediatric psychology. J Pediatr Psychol 33:983–996. 10.1093/jpepsy/jsn038 18430762 10.1093/jpepsy/jsn038 PMC2543105

[15] Revicki DA, Kleinman L, Cella D (2022) A history of health-related quality of life outcomes in psychiatry. Dialog Clin Neurosci 16:127–135. 10.31887/DCNS.2014.16.2/drevicki 10.31887/DCNS.2014.16.2/drevicki PMC4140507 25152652

[16] Megari K (2013) Quality of life in chronic disease patients. Health Psychol Res 10.4081/hpr.2013.e27 10.4081/hpr.2013.e27 PMC4768563 26973912

[17] Hernández-Segura N, Marcos-Delgado A, Pinto-Carral A, Fernández-Villa T, Molina AJ (2022) Health-Related Quality of Life (HRQOL) Instruments and mobility: a systematic review. Int J Environ Res Public Health 10.3390/ijerph192416493 10.3390/ijerph192416493 PMC9779526 36554369

[18] Alomar MJ (2014) Factors affecting the development of adverse drug reactions. Saudi Pharm J 22:83–94. 10.1016/j.jsps.2013.02.003 24648818 10.1016/j.jsps.2013.02.003 PMC3950535

[19] Kumar A, Ahuja J, Shrivastava TP, Kumar V, Kalaiselvan V (2018) Statistical signal process in R language in the pharmacovigilance programme of India. Therapeutic Innov Regul Sci 52:329–333. 10.1177/2168479017728988 10.1177/2168479017728988 29714534

[20] World Bank Group (2016) Bihar - Poverty, growth and inequality (English). India state briefs Washington, DC. http://documents.worldbank.org/curated/en/781181467989480762/Bihar-Poverty-growth-and-inequality. Accessed 1 June 2024

[21] Niti aayog (2024) National Institution for Transforming India.https://social.niti.gov.in/hlt-ranking. Accessed 1 June 2024

[22] Hartwig SC, Siegel J, Schneider PJ (1992) Preventability and severity assessment in reporting adverse drug reactions. Am J Hosp Pharm 49:2229–2232 1524068

[23] Herdman M, Gudex C, Lloyd A, Janssen MF, Kind P, Parkin D et al (2011) Development and preliminary testing of the new five-level version of EQ-5D (EQ-5D-5L). Qual Life Res 20:1727–1736. 10.1007/s11136-011-9903-x 21479777 10.1007/s11136-011-9903-x PMC3220807

[24] Kind P (1996) The EuroQoL instrument: an index of health-related quality of life. Qual life Pharmacoeconomics Clin Trials 2:191–201

[25] Kind P, Brooks R, Rabin R (2005) EQ-5D concepts and methods. A developmental history. Springer Dordrecht. 10.1007/1-4020-3712-0

[26] Jyani G, Prinja S, Garg B, Kaur M, Grover S, Sharma A et al (2023) Health-related quality of life among Indian population: the EQ-5D population norms for India. J Global Health. 10.7189/jogh.13.04018 10.7189/jogh.13.04018 PMC9936451 36799239

[27] Euro QL (2023) India value set SPSS. https://euroqol.org/wp-content/uploads/2022/03/India_valueset_SPSS.txt. Accessed 1 July 2023

[28] Jyani G, Sharma A, Prinja S, Kar SS, Trivedi M, Patro BK et al (2022) Development of an EQ-5D value set for India using an extended design (DEVINE) study: the Indian 5-level version EQ-5D value set. Value Health 25:1218–1226. 10.1016/j.jval.2021.11.1370 35779943 10.1016/j.jval.2021.11.1370

[29] Gupta N, Pandey AK, Dimri K, Jyani G, Goyal A, Prinja S (2022) Health-related quality of life among breast cancer patients in India. Support Care Cancer 30:9983–9990. 10.1007/s00520-022-07395-7 36222977 10.1007/s00520-022-07395-7

[30] Chawla S, Kumar S (2017) Adverse drug reactions and their impact on quality of life in patients on antipsychotic therapy at a tertiary care center in Delhi. Indian J Psychol Med 39:293–298 28615763 10.4103/0253-7176.207332 PMC5461839

[31] Krishnarajan D, Sivasakthi K, Ariyamol R, Kumar DN, Varghese S (2021) A prospective observational study of chemotherapy-induced adverse drug reaction and the quality of life in cancer patients in a tertiary care hospital. J Cancer Res Ther 17:530–536 34121703 10.4103/jcrt.JCRT_1015_19

[32] Kekäle M, Peltoniemi M, Airaksinen M (2015) Patient-reported adverse drug reactions and their influence on adherence and quality of life of chronic myeloid leukemia patients on per oral tyrosine kinase inhibitor treatment. Patient Prefer Adherence 1733–1740 10.2147/PPA.S92125 PMC4677761 26677317

[33] Sineke T, Evans D, Schnippel K, van Aswegen H, Berhanu R, Musakwa N, Lönnmark E, Long L, Rosen S (2019) The impact of adverse events on health-related quality of life among patients receiving treatment for drug-resistant tuberculosis in Johannesburg, South Africa. Health Qual Life Outcomes 17:1–15 10.1186/s12955-019-1155-4 PMC6545023 31151398

[34] Martini M, Di Taranto M, Höfer V, Worm M, Bilò MB (2023) Health-related quality of life and mental health in drug hypersensitivity reactions and drug-induced anaphylaxis: a systematic review and meta-analysis. J Allergy Clin Immunol Pract 10.1016/j.jaip.2023.03.012 36958518

[35] Wadasadawala T, Mohanty SK, Sen S, Khan PK, Pimple S, Mane JV et al (2023) Health-Related Quality of Life (HRQoL) using EQ-5D-5L: value set derived for Indian breast cancer cohort. Asian Pac J Cancer Prev 24:1199–1207. 10.31557/apjcp.2023.24.4.1199 10.31557/APJCP.2023.24.4.1199 PMC10352737 37116141

[36] Wong EL, Xu RH, Cheung AW (2019) Health-related quality of life among patients with hypertension: population-based survey using EQ-5D-5L in Hong Kong SAR, China. 10.1136/bmjopen-2019-032544. BMJ open 10.1136/bmjopen-2019-032544 PMC6773333 31562165

[37] Abedini MR, Bijari B, Miri Z, Shakhs Emampour F, Abbasi A (2020) The quality of life of the patients with diabetes type 2 using EQ-5D-5 L in Birjand. Health Qual Life Outcomes 18:1–9. 10.1186/s12955-020-1277-8 32000785 10.1186/s12955-020-1277-8 PMC6990543

[38] Petrova NN, Khvostikova DA (2021) Prevalence, structure, and risk factors for mental disorders in older people. Adv Gerontol 11:409–415. 10.1134/S207905702104009333993676

[39] Zhang S, Xiang W (2019) Income gradient in health-related quality of life—the role of social networking time. Int J Equity Health. 10.1186/s12939-019-0942-1. 18:1– 0 30876427 10.1186/s12939-019-0942-1 PMC6419834

[40] Thangiah G, Said MA, Majid HA, Reidpath D, Su TT (2020) Income inequality in quality of life among rural communities in Malaysia: a case for immediate policy consideration. Int J Environ Res Public Health 17:8731. 10.3390/ijerph17238731 33255397 10.3390/ijerph17238731 PMC7727827

[41] Thomson RM, Igelström E, Purba AK, Shimonovich M, Thomson H, McCartney G et al (2022) How do income changes impact on mental health and wellbeing for working-age adults? A systematic review and meta-analysis. Lancet Public Health 7:515–528. 10.1016/S2468-2667(22)00058-5 10.1016/S2468-2667(22)00058-5 PMC7614874 35660213

[42] Amin MA, Ahmed M, Nahin S, Kakoly NS (2022) Assessment of depression and anxiety among admitted people with heart disease conditions: a cross-sectional hospital-based study in a Bangladeshi population during the COVID-19. Front Psychiatry. 10.3389/fpsyt.2022.895224 10.3389/fpsyt.2022.895224 PMC9302201 35873273

[43] Poudel AN, Zhu S, Cooper N, Roderick P, Alwan N, Tarrant C et al (2021) Impact of Covid-19 on health-related quality of life of patients: a structured review. PLoS ONE. 10.1371/journal.pone.0259164 10.1371/journal.pone.0259164 PMC8553121 34710173

[44] Yavuz Daglioglu EB, Cadirci D, Aksoy M (2020) Effects of disease severity on quality of life in patients with psoriasis. Dermatol Ther. 10.1111/dth.14422 33068067 10.1111/dth.14422

[45] O’Brien EC, Hellkamp AS, Neely ML, Swaminathan A, Bender S, Snyder LD et al (2020) Disease severity and quality of life in patients with idiopathic pulmonary fibrosis: a cross-sectional analysis of the IPF-PRO registry. Chest 157:1188–1198. 10.1016/j.chest.2019.11.042 31954102 10.1016/j.chest.2019.11.042

